# High lymphocyte population-related predictive factors for a long-term response in non-small cell lung cancer patients treated with pemetrexed: a retrospective observational study

**DOI:** 10.1186/s12967-021-02761-1

**Published:** 2021-02-28

**Authors:** Issei Sumiyoshi, Takahiro Okabe, Shinsaku Togo, Haruhi Takagi, Hiroaki Motomura, Yusuke Ochi, Naoko Shimada, Mizuki Haraguchi, Rina Shibayama, Yuichi Fujimoto, Junko Watanabe, Moe Iwai, Kotaro Kadoya, Shin-ichiro Iwakami, Kazuhisa Takahashi

**Affiliations:** 1 Division of Respiratory Medicine, Juntendo University Faculty of Medicine & Graduate School of Medicine, 2-1-1 Hongo, Bunkyo-ku, Tokyo, 113-8421 Japan; 2 Leading Center for the Development and Research of Cancer Medicine, Juntendo University, Tokyo, Japan; 3 Division of Respiratory Medicine, Juntendo University Shizuoka Hospital, Shizuoka, Japan

**Keywords:** Absolute lymphocyte count, Immunogenic cell death, Neutrophil-to-lymphocyte ratio, Non-small-cell lung cancer, Pemetrexed, Programmed cell death-1, Programmed death-ligand 1

## Abstract

**Background:**

Regimens combining pemetrexed (PEM) and immune checkpoint inhibitors (ICIs) targeting programmed cell death-1 (PD-1) or programmed death-ligand 1 (PD-L1) are widely used for the treatment of advanced non-squamous non-small-cell lung cancer (NSq-NSCLC). Recently, PEM was shown to induce immunogenic cell death (ICD) and to enhance immune-regulatory genes. Some patients demonstrate an extremely long-term response to PEM. It is possible that the continued response in these patients is dependent on not only the pharmacological induction of cytotoxic cell death but also antitumor immunity. However, factors that can predict outcomes associated with long-term PEM administration using blood test results have not yet been elucidated. We investigated the clinical characteristics and predictive factors in patients with advanced NSq-NSCLC who underwent long-term PEM maintenance therapy.

**Methods:**

In total, 504 patients with advanced NSq-NSCLC who received PEM combination therapy/monotherapy (n = 414) or paclitaxel (PTX) combination therapy (n = 90) between January 2010 and November 2019 were recruited; 381 patients were retained for the final analysis. Patients treated with PEM (n = 301) were divided into subgroups according to the total cycles of PEM (≥ 17 [n = 25] for the long-term administration group and ≤ 16 [n = 276] for the intermediate/short-term group) and compared with another population (n = 80) treated with PTX combination regimen. We investigated clinical features and predictive biomarkers, focusing on immune-regulatory factors, absolute lymphocyte count (ALC), neutrophil-to-lymphocyte ratio (NLR), and PD-1 and PD-L1 expression, to predict long-term response to PEM.

**Results:**

The long-term PEM administration group exhibited a higher ALC and a lower NLR than the shorter-term group did. Both these markers displayed greater association with progression-free survival and overall survival in the PEM combination therapy group than in the PTX combination therapy group. Increased PD-1 lymphocytes were associated with the long-term PEM response group, as PD-L1 expression in tumors was associated with a high incidence of immune-related adverse effects following ICI administration.

**Conclusions:**

ALC, NLR, and PD-1 expression are PEM-mediated predictive biomarkers that are indirectly related to tumor immunity and can provide useful predictive information on the long-term response to PEM in patients with NSq-NSCLC.

**Supplementary Information:**

The online version contains supplementary material available at 10.1186/s12967-021-02761-1.

## Background

Pemetrexed (PEM), a multi-targeting antifolate antagonist, is widely established as the main chemotherapeutic agent, in combination with platinum (cisplatin or carboplatin), for first-line treatment of advanced non-squamous non-small cell lung cancer (NSq-NSCLC) [1, 2]. Moreover, PEM maintenance therapy has been demonstrated to be an efficacious strategy for patients with advanced NSq-NSCLC who do not show disease progression during PEM-platinum induction therapy [3–6]. The PARAMOUNT trial reported favorable outcomes with the PEM regimen, with a median overall survival (OS) of 16.9 months, and a median of four cycles for PEM maintenance therapy [3]. However, physicians often encounter patients who have been treated with many rounds of PEM. Although the response rate was higher than that of 9.1% observed for overall PEM monotherapy, it should be noted that some patients have received PEM for a long period without disease progression [7]. Previous studies have reported several factors that are related to the clinical outcomes of PEM: sex [8], never-smoker [9, 10], epidermal growth factor receptor (EGFR) mutation [11], anaplastic lymphoma kinase (ALK) gene rearrangement [9–14], low tumor thymidylate synthase (TS) level [15–17], thyroid transcription factor-1 (TTF-1) expression [15, 16], low serum leptin level [18], and low tumor burden [11]. In the clinical setting, some patients achieve long-term responses of more than one year (over 17 cycles of a tri-weekly regimen). Predictors of long-term PEM response that have been reported include M1a stage, lower TS expression, and smoking status [11, 19]. However, the mechanisms underlying this long-term PEM response are unclear.

Immune checkpoint inhibitors (ICIs) targeting PD-1 or PD-L1 established a new paradigm for lung cancer treatment and increased survival benefits [20, 21]. The combination therapy of PEM, platinum, and ICIs has been proposed as the first-line standard regimen for the treatment of advanced lung adenocarcinoma, irrespective of tumor PD-L1 expression [6]. Based on these positive results, we hypothesized that the combination of PEM and ICIs might have a synergistic effect on tumor immunity. The antitumor effects of ICIs are exerted by disturbing immunological tolerance; however, the association between PEM and tumor immunity has not yet been elucidated. Recent basic research has successfully shown that, PEM activates tumor immune responses and immunogenic cell death (ICD) and enhances immune-regulatory genes [1]. These findings suggest that PEM-induced tumor immunity may be involved in the mechanisms of long-term PEM responses.

However, studies investigating factors associated with immune-mediated long-term responses to PEM are limited, and most studies have analyzed the efficacy of PEM without considering the length of treatment. The present study aimed to investigate the clinical characteristics and predictive factors in patients with advanced NSq-NSCLC who were treated long-term with PEM maintenance therapy. We additionally sought to detect predictive biomarkers that reveal the association between PEM and tumor immunity in patients that have undergone long-term PEM therapy. We focused on factors that had the potential to predict the response to immunotherapy, including the neutrophil-to-lymphocyte ratio (NLR) [22–24] and absolute lymphocyte count (ALC) [25, 26], both of which have been associated with activation of antitumor immunity. We hypothesized that NLR and ALC may also be useful in predicting the long-term response to PEM and in elucidating the predictive factors of long-term PEM responses, which will help to select precision medicine for patients with advanced NSq-NSCLC.

## Methods

### Patient characteristics and study design

**Fig. 1:**
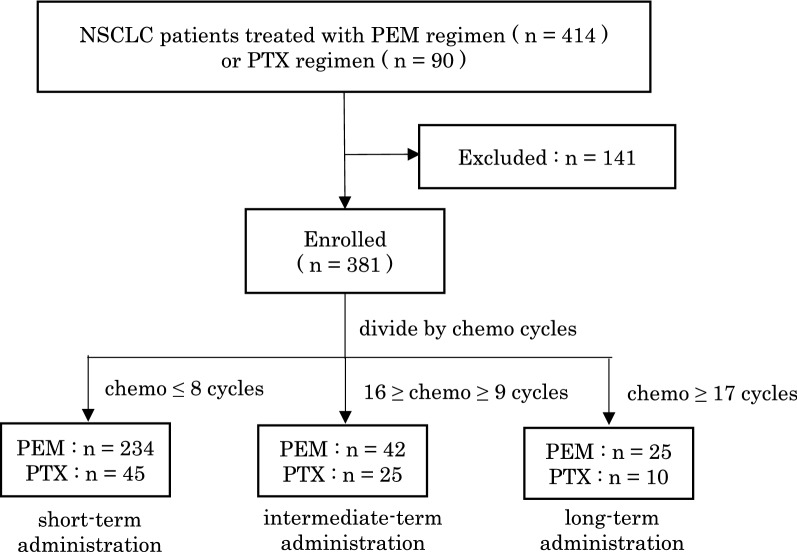
Patient enrollment process. 381 NSq-NSCLC patients treated with PEM regimen or PTX regimen were finally enrolled, divided into three groups by treatment cycles. NSCLC = Non-small cell lung carcinoma, PEM = Pemetrexed, PTX = Paclitaxel

This retrospective observational study was conducted with advanced NSq-NSCLC patients who received PEM (excluding concomitant ICIs) for the treatment of unresectable tumors, postoperative recurrence, or recurrence after curative chemoradiotherapy over the period January 2010 to November 2019. Of the 414 patients treated with PEM, 131 were excluded due to the following criteria: they were receipts of adjuvant or neoadjuvant therapy (n = 37), curable chemoradiotherapy (n = 1), or ongoing PEM treatment in November 2019 (n = 7); had missing blood cell count or white blood cell differentiation reports (n = 7); discontinued early due to adverse events such as allergic reaction (n = 21); dropped out due to relocation or self-interruption of hospital visits (n = 30); and/or received combination therapy with molecular targeted drugs (clinical trials; n = 10). In the remaining 301 cases, we performed a comparative analysis of the clinical characteristics of long-term PEM responses between the following three groups: long-term response (PEM ≥ 17 cycles; n = 25), intermediate-term response (16 cycles ≥ PEM ≥ 9 cycles; n = 42), and short-term response (PEM ≤ 8 cycles; n = 234). Additionally, for comparison, we recruited 90 patients treated with paclitaxel (PTX) + carboplatin (CBDCA) + Bev, another standard regimen during the study period, to clarify the PEM-specific predictive response markers [27, 28]. Ten patients were excluded, while the remaining 80 patients were divided into three groups and analyzed in the same way (Fig. 1).

### PD-L1 expression in tumor tissues

Tumor biopsy specimens underwent immunohistochemical staining using a monoclonal antibody against PD-L1 (22C3 pharmDx assay; Agilent Technologies, Santa Clara, California, USA) as previously described [29].

### PD-1 positive lymphocyte measurements

Out of 25 patients who received long-term PEM treatment (PEM ≥ 17 cycles), we collected blood samples from 5 patients during PEM maintenance therapy and analyzed for PD-1 expression in lymphocytes. For comparison, five patients from the intermediate/short-term administration group (PEM ≤ 16 cycles) and five patients from the control group (chemo-naïve advanced NSCLC patients) were examined. Blood samples were collected in EDTA-2Na vacuum blood collection tubes (VP-NA070KN60; Terumo Corp., Shibuya-ku, Tokyo, Japan). We diluted 1 mL of blood with an equal volume of phosphate-buffered saline (PBS), and layered it on 4 mL of differential gravity separation solution (Ficoll**®**-Paque PREMIUM 1.084; Danaher Corp., Washington, D.C., USA) in a 15-mL conical tube. Centrifuge separation was performed (900 g for 15 min, centrifuged at 25 °C), and the mononuclear cell layer was collected using a pipette and diluted in 25 mL of PBS with 2% fetal bovine serum (FBS). After centrifugation at 500 g for 5 min at 25 °C, the supernatant was removed by aspiration, and the enriched fraction of leukocytes was obtained. These procedures were performed within six hours of blood collection, and the collected leukocytes were put into a cell fixation solution (PBS with 4% paraformaldehyde-phosphate). Immediately prior to immunostaining, the leukocytes were permeabilized (0.3% TritonX-100, 10 min, 25 °C). FBS (10%) and Fc receptor blocking reagent (Miltenyi Biotec, Bergisch Gladbach, North Rhine-Westphalia, Germany) were added to block nonspecific binding (10 min, 25 °C). Leukocytes were subsequently stained with an antibody reagent cocktail containing 4′,6-diamidino-2-phenylindole (DAPI), Alexa Fluor 647 labeled anti-human cluster of differentiation (CD) 45 antibody (clone HI30; Biolegend, San Diego, California, USA), Phycoerythrin (PE) labeled anti-human PD-1 antibody (clone EH12.1; BD Biosciences, San Jose, California, USA), and FcR blocking reagent (30 min, 25 °C). For staining of negative control samples, isotype control antibodies were used instead of PE-labeled anti-human PD-1 antibodies. Unreacted antibodies were washed out with PBS containing 2% FBS. After fluorescence immunostaining, flow cytometry was performed within 3 h using a BD FACSAria™ Fusion Cell Sorter (BD biosciences) and the analysis was performed using FlowJo™ software (BD biosciences).

In the analysis, lymphocytes were identified by a low forward scatter (FSC) area and low side scatter (SSC) area gate. Doublet discrimination was performed using FSC-H (height), FSC-W (width), SSC-H, and SSC-W. We gated the fractions that were positive for DAPI to include nucleated cells. Finally, we expanded the PE vs. Alexa Fluor 647 plot and identified the double-positive fraction as PD-1-positive lymphocytes. The gates for the positive fraction of PD-1 were determined by comparison with the isotype control data.

### Statistical analysis

We used one-way analysis of variance (ANOVA) and Tukey's test to compare the three groups. Unpaired t-test (Mann–Whitney test) or chi-square test was used for two-group comparisons. Binomial logistic regression was used for performing univariate/multivariate analyses. Progression-free survival (PFS) and OS are shown as Kaplan–Meier curves, and P values were calculated using the log-rank method. Hazard ratios (HRs) were calculated using Cox regression analysis. Statistical analysis was performed using IBM SPSS version 25.0 (IBM Corp., Armonk, New York, USA) and graphs were drawn in GraphPad Prism 8.2.1. (GraphPad Software Inc., San Diego, California, USA). All experiments were approved by the ethics committee of Juntendo University School of Medicine (Approval No. 19–135, 860).

## Results

### Patient characteristics

Patient characteristics are shown in Table 1. Among the 414 patients treated with PEM, 301 were finally enrolled. Their median age was 66 years, and 173 (57.5%) patients were male. Performance status 0/1/2 was seen in 106/159/36 patients, respectively. Stages III and IV were seen in 23 and 199 patients, respectively. Recurrence after definitive treatment (operation or chemoradiation) was seen in the remaining patients (n = 77).

**Table 1:** Clinical and demographic features of study subjects

	PEM ± Platinum ± Bev ( n = 301)	PTX + CBDCA + Bev ( n = 80)	P value
Age	66 [39–92]	64 [36–79]	0.025 *
Sex male/female	172/129	54/26	0.094
BMI	21.9 [14.6–33.0]	22.4 [15.2–30.2]	0.137
Smoking history			
Never/past/current	106/122/73	25/33/22	0.754
Brinkman’s index	400 [0–920]	400 [0–3600]	0.921
ECOG PS 0/1/2	106/159/36	40/36/4	0.026 *
Pathological diagnosis			
Adenocarcinoma/others	287/14	75/5	0.559
Stage (8th edition)			
IIIB/IIIC/IVA/IVB/Rec/unknown	19/4/110/89/77/2	11/2/26/18/23/0	0.208
Driver mutation			
EGFR ± /unknown	87/200/14	27/49/4	0.679
ALK ± /unknown	15/227/59	3/56/21	0.410
Therapy line			
1st line/2nd line/others	179/83/39	60/14/6	0.038 *

The data and variabilities are presented as median and range

*PEM* Pemetrexed, *Platinum* Cisplatin or Carboplatin, *PTX* Paclitaxel, *CBDCA* Carboplatin, *Bev* Bevacizumab, *BMI* body mass index, *ECOG PS* Eastern Cooperative Oncology Group Performance Status, *Rec* recurrence after definitive treatment (operation or chemoradiation), *EGFR* epidermal growth factor receptor, *ALK* anaplastic lymphoma kinase. *: P < 0.05 (Unpaired t-test, Chi-square test)

Additionally, we compared PEM-based regimens with another standard regimen during the study period, PTX + CBDCA + Bev, to clarify the PEM-specific predictive response markers [27, 28]. Among the 90 patients treated with PTX + CBDCA + Bev, 80 were enrolled. Age, ECOG-PS, and therapy line of PTX-treated patients were significantly lower than those of PEM-treated patients (P < 0.05).

### Monitoring of leukocyte fractionation during administration of PEM

**Fig. 2:**
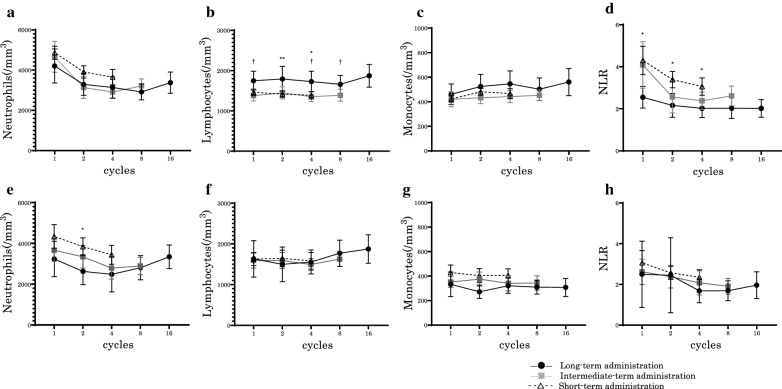
Transitions in blood cell counts. **a**–**d;** Transitions in neutrophil (**a**), lymphocyte (**b**), monocyte (**c**) counts and neutrophil-to-lymphocyte ratio (**d**) in the peripheral blood of patients treated with Pemetrexed ± Platinum ± Bevacizumab. The mean value of lymphocyte counts is significantly higher in the long-term administration group. **e–h;** Transitions in neutrophil (**e**), lymphocyte (**f**), monocyte (**g**) counts and neutrophil-to-lymphocyte ratio (**h**) in the peripheral blood of patients treated with Paclitaxel + Carboplatin + Bevacizumab. There was no significant difference. NLR = neutrophil–lymphocyte ratio. Data are presented as mean ± 95%CI. *: Long-term administration vs. short-term administration; P < 0.05 (Unpaired t-test)**.** **: Long-term administration vs. short-term administration; P < 0.01 (Unpaired t-test)**.** †: Long-term administration vs. Intermediate-term administration; P < 0.05 (Unpaired t-test)

We examined the transitions of 301 PEM-treated patients in peripheral blood leukocyte fractionation and the NLR at the following PEM administration time points: 1st (pretreatment), 2nd, 4th, 8th, and 16th cycles. ALCs were statistically higher in the long-term administration group throughout the entire treatment period than in the other groups (Fig. 2b). There was a small tendency toward lower neutrophil and higher monocyte levels in the long-term administration group, but the difference was not statistically significant (Fig. 2a, c). However, NLR was significantly higher in the short-term administration group throughout the entire treatment period compared to the long-term administration group (Fig. 2d). Otherwise, transitions of leukocyte fractionation of the PTX-based regimen were not significantly different in the three groups, except for neutrophil at the time point of 2nd cycle (Fig. 2e–h). To examine the independent factors of long-term PEM response, we additionally performed multiple logistic regression analysis. Age, sex, performance status, smoking history, stage according to 8th edition of the TNM classification, line of therapy, platinum combination, blood cell counts, and NLR were used as variables. Age, stage, ALC, and NLR were significantly related to the long-term PEM response in the univariate analysis (p < 0.05). Age, stage, and blood cell counts were identified as independent factors related to the long-term PEM response in the multivariate analysis (Table 2).

**Table 2:** Logistic regression analysis for prognostic factors of long-term (≧17 courses) response of pemetrexed

Characteristics	Univariate analysis		Multivariate analysis	
	P value	Hazard ratio (95%CI)	P value	Hazard ratio (95%CI)
Age	0.006*	1.067 (1.018–1.118)	0.027*	1.068 (1.007–1.132)
Sex	0.588	0.797 (0.351–1.810)	0.125	0.413 (0.133–1.279)
ECOG PS	0.228	3.485 (0.457–26.580)	0.459	0.441 (0.051–3.851)
Smoking history	0.425	0.691 (0.279–1.713)	0.261	0.492 (0.143–1.694)
Stage (8th edition)	0.013*	0.656 (0.470–0.914)	0.030*	0.666 (0.461–0.962)
Therapy line	0.630	1.232 (0.526–2.887)	0.876	0.918 (0.313–2.690)
Platinum combination	0.463	1.378 (0.585–3.247)	0.764	0.841 (0.272–2.604)
Neutrophils (/mm^2^)	0.228	1.000 (1.000–1.000)	0.041*	1.000 (0.999–1.000)
Lymphocytes (/mm^2^)	0.020*	1.000 (1.000–1.001)	0.033*	1.001 (1.000–1.001)
Monocytes (/mm^2^)	0.337	1.001 (0.999–1.003)	0.016*	1.001 (1.001–1.007)
NLR	0.023*	0.693 (0.505–0.951)	※	※

*ALC* absolute lymphocyte count (/mm^3^), *ECOG PS* Eastern Cooperative Oncology Group Performance Status, *NLR* Neutrophil-to-Lymphocyte ratio, *95%CI* 95% confidence interval

*: P < 0.05 (Binomial logistic regression)

※: Not available due to multi-collinearity

### Association of leukocyte fractionation with clinical outcomes in PEM-based regimens

**Fig. 3:**
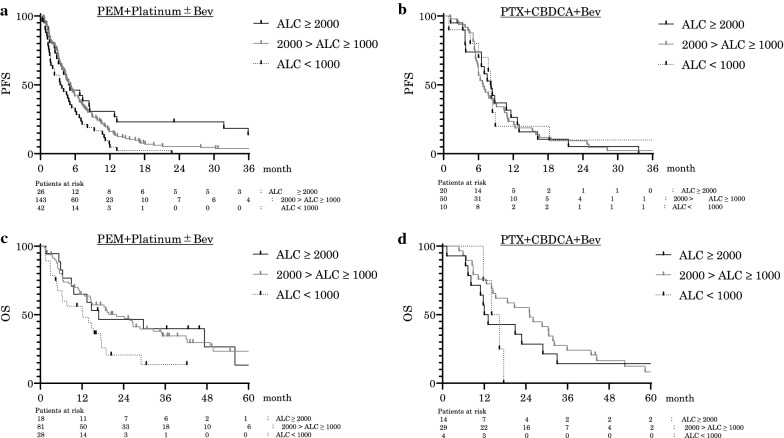
Correlation between pretreatment ALC and prognosis of chemotherapy. Kaplan–Meier PFS curves of PEM + Platinum ± Bev (**a**), PTX + Platinum ± Bev (**b**), and Kaplan–Meier OS curve of first-line PEM + Platinum ± Bev (**c**), PTX + Platinum ± Bev (**d**) are shown. Patients were divided into three groups: high ALC group (ALC ≥ 2000), intermediate ALC group (2000 > ALC ≥ 1000), and low ALC group (ALC < 1000). High ALC is related to better prognosis of PEM + Platinum ± Bev. PFS = progression-free survival, ALC = absolute lymphocyte count (/mm^3^), PEM = Pemetrexed, PTX = Paclitaxel, Platinum = Cisplatin or Carboplatin, Bev = Bevacizumab

We tested whether the pretreatment ALC could serve as a sensitive and specific predictive biomarker for PEM combination therapy compared to other standard combination therapies in a clinical setting. Of the 301 patients, 211 patients who received the PEM + platinum ± Bev regimen were enrolled in the analysis and classified into one of the following three groups: high ALC (ALC ≥ 2000), intermediate ALC (2000 > ALC ≥ 1000), and low ALC (ALC < 1000) with the median PFS as 4.9, 5.2, and 3.4 months, respectively. The median PFS was statistically longer in the high ALC group (P = 0.010, HR: 0.496, 95% CI 0.296–0.829) and the intermediate ALC group (P = 0.024, HR: 0.672, 95% CI 0.474–0.952) in patients on the PEM + platinum ± Bev regimen (Fig. 3a).

We enrolled 117 PEM-treated patients who were receiving 1st line treatment and were driver mutation negative for OS analysis. The median OS was 16.8, 21.9, and 12.2 months, respectively, and tended to be longer in the high ALC group (P = 0.065, HR: 0.500, 95% CI 0.244–1.027), and was significantly longer in the intermediate ALC group (P = 0.012, HR: 0.517, 95% CI 0.308–0.866) in patients treated with the PEM + platinum ± Bev regimen (Fig. 3c). For comparison, the 80 patients who received PTX + CBDCA + Bev were analyzed among the three groups (high, intermediate, and low ALC groups). The median PFS was 4.9, 5.2, and 3.4 months, respectively (Fig. 3b). Among the 47 patients treated with 1st line therapy who were driver mutation negative, the median OS was 12.6, 25.1, and 15.3 months, respectively. There were no statistically significant differences between any of the groups in the PTX + CBDCA + Bev regimen (Fig. 3d). High white blood cell (WBC) and neutrophil counts before treatment tended to be associated with a worse prognosis. The median PFS in the high and low WBC groups were 3.2 and 5.3 months, respectively, and were statistically longer in the high WBC group (P = 0.006, HR: 0.613, 95% CI 0.429–0.875) in patients who received the PEM + platinum ± Bev regimen (Additional file 1: Figure S1a). The median PFS in patients with high and low total neutrophil counts was 3.3 and 5.2 months, respectively, and the patients with a high neutrophil count group showed a tendency towards a longer PFS, although the difference was not statistically significant (P = 0.067, HR: 0.685, 95% CI 0.456–1.030; Additional file 1: Figure S1b). There were no statistically significant differences in monocyte counts (Additional file 1: Figure S1c).

### Association of NLR with clinical outcomes in PEM-based regimens

**Fig. 4:**
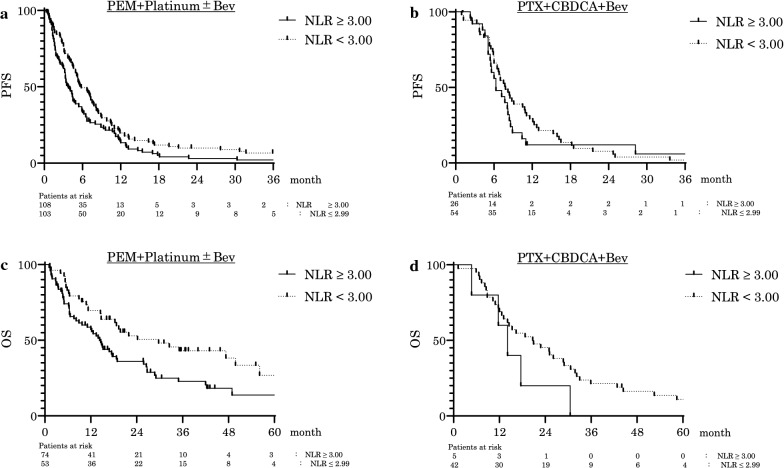
Correlation between pretreatment NLR and prognosis of chemotherapy. Kaplan–Meier PFS curves of PEM + Platinum ± Bev (**a**), PTX + Platinum ± Bev (**b**), and Kaplan–Meier OS curve of first-line PEM + Platinum ± Bev (**c**), PTX + Platinum ± Bev (**d**) are shown. Patients were divided into two groups: high NLR group (NLR ≥ 3.00) and low NLR group (NLR < 3.00). Low NLR is related to better prognosis with both regimens. PFS = progression-free survival, NLR = neutrophil-to-lymphocyte ratio, PEM = Pemetrexed, PTX = Paclitaxel, Platinum = Cisplatin or Carboplatin, Bev = Bevacizumab

Similarly, we verified whether the NLR accurately reflects the therapeutic effects of chemotherapy. We recruited 211 patients who received PEM + platinum ± Bev with the same criteria as in the ALC analyses and divided them into two groups: high NLR group (NLR ≥ 3.00) and low NLR group (NLR < 3.00). The median PFS (months) was 3.8 and 5.8, respectively, and the low NLR group had a significantly longer PFS (P = 0.026, HR: 0.725, 95% CI 0.547–0.962; Fig. 4a). Among the 117 patients who were receiving treatment as 1st line therapy and who were driver mutation negative, the median OS was 14.3 and 29.7 months, respectively, and the median OS was significantly longer in the low NLR group (P = 0.015, HR: 0.588, 95% CI 0.384–0.900; Fig. 4c).

Otherwise, for the 80 patients who received PTX + CBDCA + Bev, the median PFS was 6.3 months (high NLR group) and 7.8 months (low NLR group), with no statistically significant difference (p = 0.456; Fig. 4b). Among the 47 patients treated with 1st line therapy who were driver mutation negative, the median OS in the low and high NLR groups were 14.1 and 20.8, respectively, with no statistically significant difference (p = 0.152; Fig. 4d).

### PD-L1 expression levels in tumor tissues in the long-term PEM administration group

**Fig. 5:**
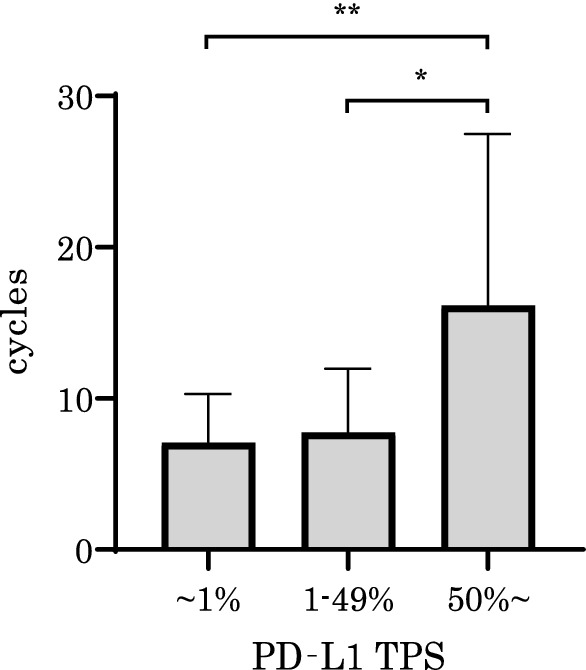
Tissue expression of PD-L1 and PEM treatment cycles. Positive correlation between PD-L1 TPS and PEM cycles were revealed. PEM = Pemetrexed, PFS = progression-free survival, TPS = tumor proportion score. **: P < 0.05 (Unpaired t-test) *: P < 0.1 (Unpaired t-test)

We measured the percentage of PD-L1 expression in tumor tissues from 63 patients treated with PEM + platinum ± Bev by bronchoscopic biopsy or pneumonectomy. They were categorized according to the following three groups: high PD-L1 expression (PD-L1 TPS ≥ 50%), low PD-L1 expression (49% ≥ PD-L1 TPS ≥ 1%) and no PD-L1 expression (PD-L1 TPS < 1%). The relationship between the number of PEM treatment cycles and PD-L1 expression levels was then analyzed. The number of PEM treatment cycles was significantly higher in the high PD-L1 expression group than in the no PD-L1 expression group (P = 0.031; Fig. 5). The Kaplan–Meier curves showed a trend toward better PFS in the higher PD-L1 expression group, but the difference was not statistically significant (Additional file 1: Figure S2).

### Measurement of PD-1 lymphocytes in the long-term PEM administration group

**Fig. 6:**
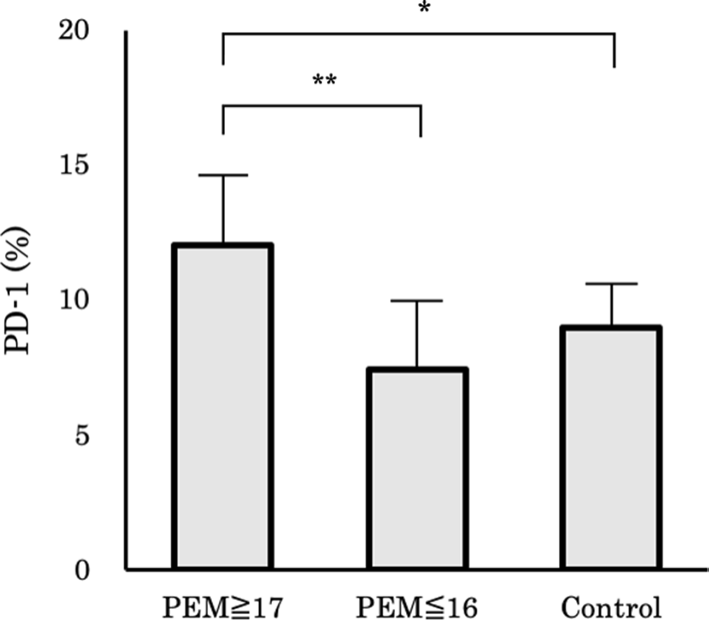
PD-1-positive lymphocytes and PEM treatment**.** PD-1-positive peripheral blood cells of long-term PEM treatment group (PEM ≥ 17 cycles), short or intermediate group (PEM ≤ 16 cycles), and control group (chemo-naive). The percentage of PD-1-positive lymphocytes was significantly high in the long-term PEM treatment group. PEM = Pemetrexed, PD-1 = programmed cell death 1. **: P < 0.05 (Unpaired t-test) *: P < 0.1 (Unpaired t-test)

We measured PD-1 expression in the peripheral blood lymphocytes of patients who had undergone long-term administration (n = 5) or intermediate/short-term administration (n = 5) of PEM. An additional five chemo-naïve patients were also included as a control group. The mean levels of PD-1 expression in the peripheral blood lymphocytes were significantly higher in the long-term PEM group than in the intermediate/short-term PEM group (P = 0.036; Fig. 6).

### Effect of preceding PEM on ICI treatment

To determine the effect of previous PEM treatment on immunotherapy outcomes, we analyzed 82 patients who received ICIs after undergoing PEM-related regimens. PFS was not significantly different regardless of previous PEM treatment cycles (Table 3). However, the frequency of immune-related adverse events (any grades) was significantly higher in the group who had undergone long-term administration of PEM compared to the other groups (45.5% vs. 18.3%, P = 0.043; Table 4).

**Table 3:** Treatment effect of immune checkpoint inhibitors after pemetrexed treatment failure

Efficacy	Long-term administration ( n = 11)	Intermediate-term administration ( n = 16)	Short-term administration ( n = 55)
Cycles	6.1 ± 8.5	5.6 ± 3.2	6.2 ± 8.3
PFS (month)	5.5 ± 6.9	3.8 ± 2.7	3.4 ± 4.4

Data are presented as mean ± SD

*PFS* progression free survival

**Table 4:** Adverse effects (any grades) of immune checkpoint inhibitors after pemetrexed treatment failure

Adverse effects	Long-term administration ( n = 11)	Intermediate-term administration ( n = 16)	Short-term administration ( n = 55)
irAE	5 (45.5%) *	3 (18.8%)	10 (18.2%)
ILD	4 (36.3%)	2 (12.5%)	2 (3.6%)
Others	1 (9.1%)	1 (6.3%)	9 (16.4%)

*irAE* immune-related adverse events, *ILD* interstitial lung disease

^*^: P < 0.05 (Chi-square test)

## Discussion

In this study, we retrospectively investigated the clinical features and predictive immune-related biomarkers in patients with advanced NSq-NSCLC treated with long-term PEM therapy. Patients in the long-term PEM administration group were significantly more likely to have high ALC and low NLR compared to the baseline during the overall course of treatment, and these were associated with long PFS and OS in patients who received PEM combination therapy. Otherwise, these predictive markers were not adaptable for PTX + CBDCA + Bev. Notably, the association between long-term PEM responses and immune checkpoint markers, high PD-L1 expression in tumor tissues, and increased percentage of PD-1 positive lymphocytes suggests that PEM may have the ability to induce not only cytotoxic death of cancer cells but also ICD.

Chemotherapy-driven ICD activates the immunogenic signals known as “damage-associated molecular patterns” (DAMPs). Naïve T-cells are primed through the cross-presentation of tumor antigens released from dying tumor cells, initiating adaptive immune responses against tumors [30]. DAMP-induced immune activators, high-mobility group box 1 (HMGB1) protein [31], and type I interferon binding to receptors on lymphocytes all contribute to the recruitment, activation, homing, antigen uptake, and maturation of activated dendritic cells. Schaer et al. demonstrated that PEM treatment alone increased T-cell activation and interferon-gamma pathway activation, as well as increased HMGB1 protein -induced dendritic cell maturation and induced immune-related gene expression in tumor tissues. However, the addition of carboplatin to the PEM regimen appeared to reduce these immunomodulatory effects. These findings support those of the present study and suggest that the high ALC population associated with long-term PEM-specific responses are the result of T-cell–intrinsic mechanisms induced by PEM. PTX chemotherapy has also been reported to induce ICD-associated DAMPs and T-cell infiltration in ovarian tumors of responsive patients, suggesting that PTX also relies upon the activation of antitumor immunity [32]. However, PTX alone and in combination with carboplatin appears to reduce the immunomodulatory effects compared to PEM alone, suggesting the existence of PEM-specific immune regulation [1].

The present results show that the baseline ALC was not associated with clinical outcomes in patients treated with PTX combination regimens. NLR is considered a useful indicator for predicting poor response to treatment in lung cancer patients. NLR is associated with shorter survival in advanced NSCLC patients treated with systemic therapy, a pattern that is consistently observed regardless of whether the treatment involves cytotoxic anticancer drugs, ICIs, or molecular target drugs [22–24, 33, 34]. The present results demonstrated a reduced NLR in the long-term PEM group, but similar results were not obtained in patients treated with a PTX combination regimen.

High PD-L1 expression in advanced lung adenocarcinoma patients treated with PEM is reportedly associated with prolonged PFS [35] and the present results demonstrated a similar trend, although it was not statistically significant because of the small number of patients in the present study. Lung cancer patients who have increased peripheral PD-1-positive CD8-positive T cells respond to anti-PD-1 antibodies [36]. The present results demonstrated that high PD-L1 expression in tumor tissues and high PD-1 lymphocyte populations were related to long-term PEM responses, as well as to a high incidence of immune-related adverse events in patients treated with ICI therapy after long-term administration of PEM. Thus, immunoreactivity to PEM may have the ability to affect the ICI response.

The present study has several limitations. First, it had a retrospective design and patients did not receive treatment according to an established protocol, which led to differences in the treatment regimens. Second, the exclusion criteria were designed to allow for accurate analysis of changes in blood cell populations, as well as PFS and OS. This resulted in the small number of patients that were enrolled in the long-term PEM response group and the PTX + CBDCA + Bev treated group and for whom we obtained PD-L1/PD-1 measurements. A larger number of patients from multiple centers are needed to provide more significant results. Third, patients treated with combination therapy of PEM and ICIs were excluded from this study to clarify the immunologic effects of PEM alone. Synergistic effects of PEM and ICIs should also be investigated in the future.

## Conclusions

Here, we demonstrated that ALC, NLR, and PD-L1/PD-1 are biomarkers of PEM-related indirect tumor immunity and are associated with clinical outcomes. These may be considered as predictive biomarkers of the long-term response to PEM, and thus, can provide useful information for clinical decision-making regarding therapeutic options for the treatment of patients with advanced non-squamous NSCLC.

## Supplementary Information

**Additional file 1: Figure S1.** Pretreatment white blood cell counts and PEM response. Kaplan-Meier PFS curves of two groups divided by WBC (**a**), neutrophil (**b**), and monocyte (**c**). High WBC and high NLR significantly correlate with shorter PFS. Abbreviations: PFS = progression-free survival, WBC = peripheral white blood cell counts, NEUT = peripheral neutrophil cell counts, MONO = peripheral monocyte cell counts, NLR = neutrophil-to-lymphocyte ratio. **Figure S2.** Tissue expression of PD-L1 and PEM response. Kaplan-Meier PFS curves were not significantly different between PD-L1 TPS high group (TPS≥50%), low group (49%≥TPS≥1%), and negative group (TPS<1%). Abbreviations: PEM = Pemetrexed, PFS = progression-free survival, TPS = tumor proportion score.

## Data Availability

The datasets generated and/or analyzed during the current study are available from the corresponding author on reasonable request.

## Abbreviations

ALC: Absolute lymphocyte count
ANOVA: Analysis of variance
CD: Cluster of differentiation
DAMP: Damage-associated molecular patterns
FBS: Fetal bovine Serum
FSC: Forward Scatter
ICD: Immunogenic cell death
ICI: Immune checkpoint inhibitors
NLR: Neutrophil-to-lymphocyte ratios
NSq-NSCLC: Non-squamous non-small-cell lung cancer
OS: Overall survival
PBS: Phosphate-buffered saline
PD-1: Programmed death cell 1
PD-L1: Programmed death ligand 1
PE: Phycoerythrin
PEM: Pemetrexed
PFS: Progression-free survival
PTX: Paclitaxel
SSC: Side scatter
WBC: White blood cell
