# Supplementary material for: High lymphocyte population-related predictive factors for a long-term response in non-small cell lung cancer patients treated with pemetrexed: a retrospective observational study
Source: J Transl Med. 2021 Feb 28;19:92. doi: 10.1186/s12967-021-02761-1 (PMC7916269; doi:10.1186/s12967-021-02761-1)
Supplement: Supplementary file 1 — Additional file 1: Figure S1. Pretreatment white blood cell counts and PEM response. Kaplan-Meier PFS curves of two groups divided by WBC (a), neutrophil (b), and monocyte (c). High WBC and high NLR significantly correlate with shorter PFS. Abbreviations: PFS = progression-free survival, WBC = peripheral white blood cell counts, NEUT = peripheral neutrophil cell counts, MONO = peripheral monocyte cell counts, NLR = neutrophil-to-lymphocyte ratio. Figure S2. Tissue expression of PD-L1 and PEM response. Kaplan-Meier PFS curves were not significantly different between PD-L1 TPS high group (TPS≥50%), low group (49%≥TPS≥1%), and negative group (TPS<1%). Abbreviations: PEM = Pemetrexed, PFS = progression-free survival, TPS = tumor proportion score. [file 12967_2021_2761_MOESM1_ESM.pptx]

## Slide 1
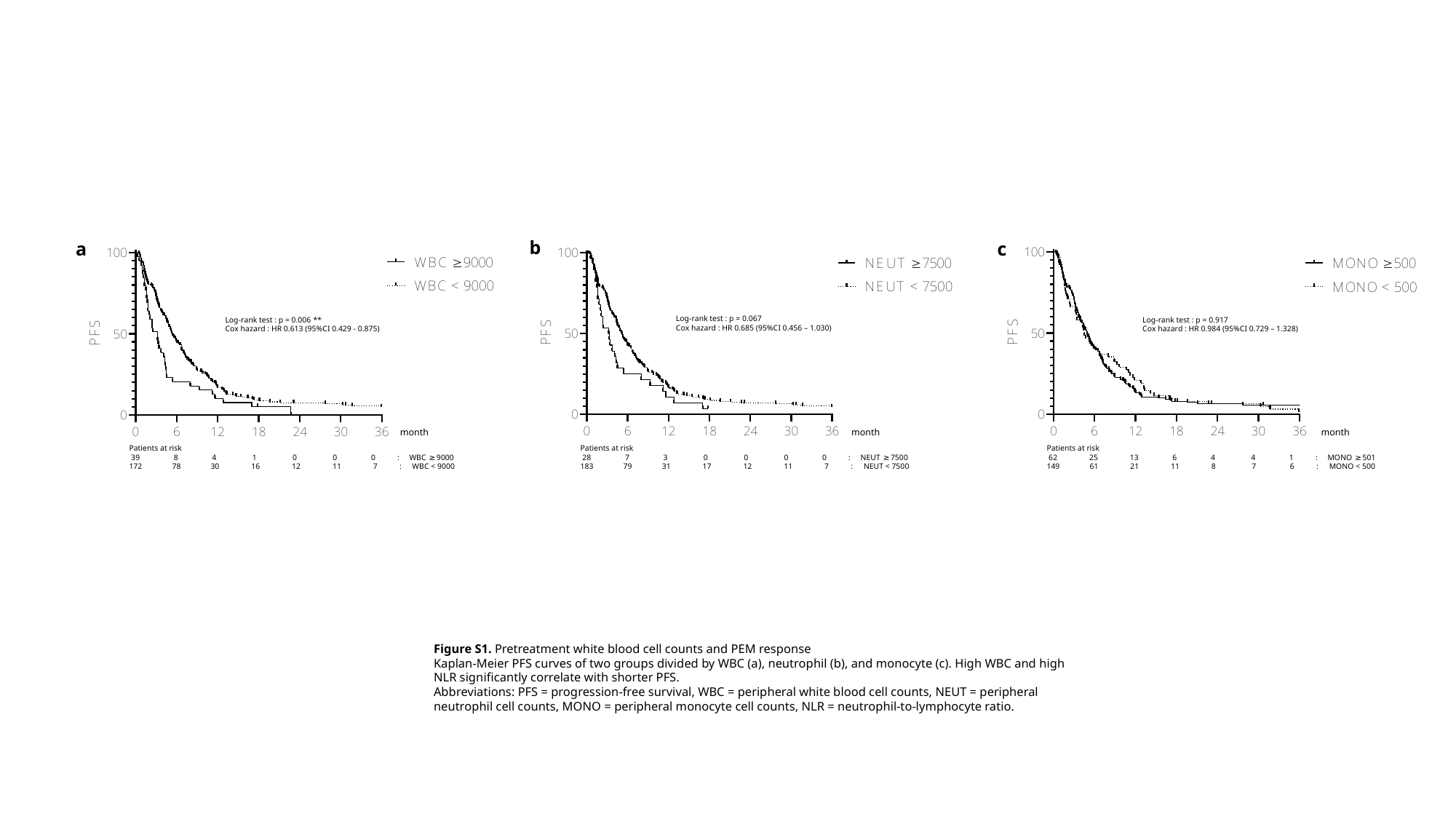

b
c
a
Log-rank test : p = 0.067
Cox hazard : HR 0.685 (95%CI 0.456 – 1.030)
Log-rank test : p = 0.917
Cox hazard : HR 0.984 (95%CI 0.729 – 1.328)
Log-rank test : p = 0.006 **
Cox hazard : HR 0.613 (95%CI 0.429 - 0.875)
month
month
month
Patients at risk
 39 8 4 1 0 0 0 : WBC ≥ 9000
172 78 30 16 12 11 7 : WBC < 9000
Patients at risk
 28 7 3 0 0 0 0 : NEUT ≥ 7500
183 79 31 17 12 11 7 : NEUT < 7500
Patients at risk
 62 25 13 6 4 4 1 : MONO ≥ 501
149 61 21 11 8 7 6 : MONO < 500
Figure S1. Pretreatment white blood cell counts and PEM response
Kaplan-Meier PFS curves of two groups divided by WBC (a), neutrophil (b), and monocyte (c). High WBC and high NLR significantly correlate with shorter PFS.
Abbreviations: PFS = progression-free survival, WBC = peripheral white blood cell counts, NEUT = peripheral neutrophil cell counts, MONO = peripheral monocyte cell counts, NLR = neutrophil-to-lymphocyte ratio.

## Slide 2
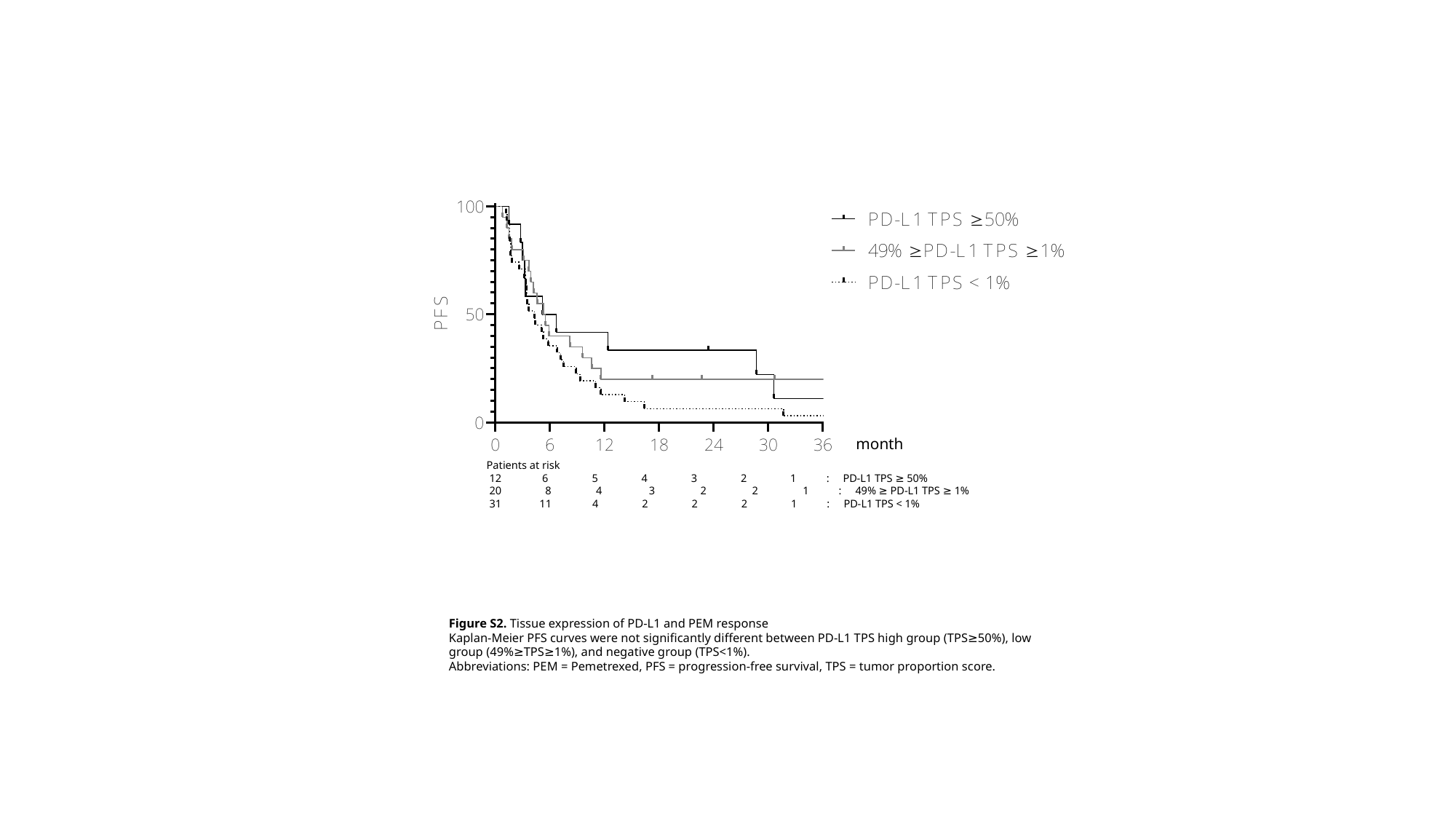

month
Patients at risk
 12 6 5 4 3 2 1 : PD-L1 TPS ≥ 50%
 20!!!!11!11 8 11 4 !!!0!!111!3 !000 2 !!!! 11 2 01 1 : 49% ≥ PD-L1 TPS ≥ 1%
 31 11 4 2 2 2 1 : PD-L1 TPS < 1%
Figure S2. Tissue expression of PD-L1 and PEM response
Kaplan-Meier PFS curves were not significantly different between PD-L1 TPS high group (TPS≥50%), low group (49%≥TPS≥1%), and negative group (TPS<1%).
Abbreviations: PEM = Pemetrexed, PFS = progression-free survival, TPS = tumor proportion score.
